# Challenges and opportunities to penetrate the blood-brain barrier for brain cancer therapy

**DOI:** 10.7150/thno.69682

**Published:** 2022-06-06

**Authors:** Dannielle H. Upton, Caitlin Ung, Sandra M. George, Maria Tsoli, Maria Kavallaris, David S. Ziegler

**Affiliations:** 1 Children's Cancer Institute, Lowy Cancer Research Centre, UNSW Sydney, Sydney, NSW, 2031, Australia.; 2 School of Women's and Children's Health, Faculty of Medicine and Health, UNSW Sydney, Sydney, NSW, 2052, Australia.; 3 Australian Centre for Nanomedicine, UNSW Sydney, Sydney, NSW 2052, Australia.; 4 Kids Cancer Centre, Sydney Children's Hospital, Randwick, NSW 2031, Australia.

**Keywords:** brain tumors, CNS malignancies, blood brain barrier, drug delivery systems, BBB disruption

## Abstract

Despite significant advances in research, the prognosis for both primary and secondary brain cancers remains poor. The blood-brain barrier (BBB) is a complex and unique semi-permeable membrane that serves as a protective structure to maintain homeostasis within the brain. However, it presents a significant challenge for the delivery of therapeutics into the brain and tumor. Some brain tumors are known to compromise BBB integrity, producing a highly heterogeneous vasculature known as the blood-tumor-barrier (BTB). Identifying strategies to bypass these obstacles to improve the penetrability of anticancer therapeutics has been the focus of research in this area. In this review, we discuss the strategies that have been investigated to evade or alter the cellular and molecular barriers of both the BBB and the BTB and detail the methods currently under preclinical or clinical investigation, including molecular, biological, and physical processes to overcome the BBB or BTB. Increased understanding of the BBB and BTB and the current methods of overcoming these barriers will enable the development of new and more effective treatment strategies for brain tumors.

## Introduction

### The Blood-Brain barrier

**Figure 1:**
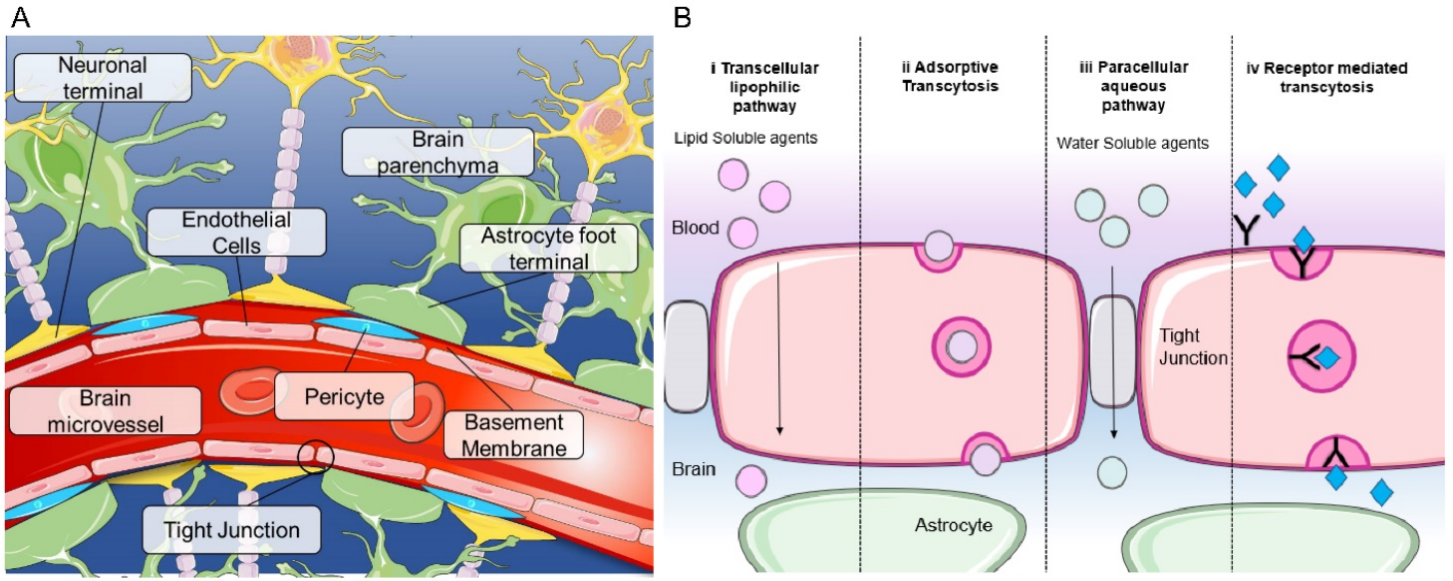
** Blood brain barrier structure and mechanisms of transport (A)** Structure of the blood brain barrier depicting brain microvessels composed of: pericytes, endothelial cells, astrocytes and neurons. **(B)** Mechanisms of transport across the BBB including (i) transcellular lipophilic pathway, (ii) adsorptive transcytosis, (iii) paracellular aqueous pathway and (iv) receptor mediated transcytosis. Image was created with BioRender.

The blood-brain barrier (BBB) is a complex and unique semi-permeable membrane that serves as a protective structure to maintain homeostasis within the brain 1. Composed of around a hundred billion capillaries, the BBB spans 20 m^2^ in the human brain and is approximately 600 kilometers long. Each capillary is approximately 7.5 μm in diameter, allowing for blood supply within 10μm of each brain cell 2. This selective physical barrier prevents the passage of molecules greater than 400 Daltons from the bloodstream into the brain, thereby protecting the microenvironment of the brain from potentially harmful exogenous substances 1. The delivery of oxygen into the brain and removal of carbon dioxide and metabolites is regulated by the BBB. The BBB cerebrovasculature is composed of a specialized endothelial cell monolayer supported by pericytes and astrocytes creating a tight interconnectivity and a unique barrier not replicated in any other organ (Figure 1A). Vascular pericytes surround 30% of the endothelial layer, controlling the vessels' diameter and regulating endothelial cell BBB-specific gene expression 2, 3. Perivascular astrocytic end-feet attached to the external wall of the endothelium upregulate BBB properties and strengthen tight junctions 4. Tight junctions between adjacent vascular endothelial cells function to restrict paracellular movement and favor transcellular movement 1 (Figure 1B). Small, lipophilic molecules diffuse passively into the brain, whereas larger hydrophilic molecules such as peptides or proteins require transport mechanisms (Figure 1B) 5. The continuity of the tight junctions, coupled with lack of fenestrae and efflux transporters, results in the BBB with distinct luminal and abluminal compartments for strict regulation and control between the blood and the brain 5.

The expression of transport proteins and efflux transporters in the BBB blocks the efficacy of therapies used to treat brain tumors. Molecular size and lipophilicity determine each therapeutic's ability to enter the brain. Moreover, once in the brain, the drug must remain active and at pharmacologically therapeutic concentrations. It is known that 100% of large molecule therapeutics and 98% of all small molecule therapeutics are excluded from the brain by the BBB 6. Hence bypassing the BBB to improve drug penetration and delivery is important for treating brain malignancies and metastatic extracranial tumors.

### The Blood-tumor barrier

Tumors are known to compromise BBB integrity, producing a highly heterogeneous vasculature known as the blood-tumor barrier (BTB). The BTB is formed by brain tumor capillaries and is distinct from the BBB, characterized by non-uniform permeability and active efflux of molecules. The BTB encompasses both existing and newly formed blood vessels that deliver nutrients and oxygen to the tumor and facilitate cell migration to other parts of the brain and is considered in many cases to be 'leakier' than the BBB 7. The BBB/BTB structural integrity is heterogenous between metastatic lesions and different primary tumor types. This leads to heterogeneous permeability, and due to these “leakier” and semi-dysfunctional vessels, water and metabolic waste are retained in the neuro-parenchymal space, increasing the interstitial and intracranial fluid pressure.

**Figure 2:**
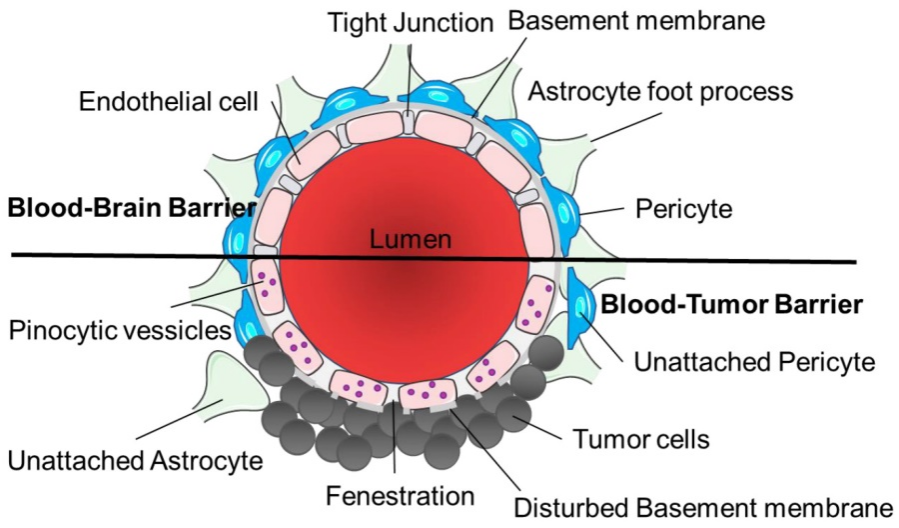
** Structural differences between the blood-brain barrier and blood-tumor barrier.** The architecture of the blood-brain barrier includes a non-fenestrated endothelial cell monolayer with tight junctions, contact with astrocyte foot processes and pericytes and a functional basement membrane. Tumor cells disrupt the normal vasculature to form the blood-tumor barrier causing fenestrations in the endothelial cells, disturbed basement membrane and unattached astrocytes and pericytes, with increased pinocytic vesicles. Image was created with BioRender.

The BTB has three distinct microvessel populations; continuous capillaries, fenestrated capillaries, and capillaries containing inter-endothelial gaps (Figure 2). Abnormal pericyte distribution, loss of astrocytic end-feet and neuronal connections are characteristic of the BTB (Figure 2) 7. Tumor cells can dislodge astrocytic end-feet, thereby disrupting BBB integrity 7. Circulating immune cells such as T-cell subpopulations and peripheral monocytes are detected in brain tumors, demonstrating the permeability of the neurovascular unit to these cell types. BTB endothelial cells have reduced junctional proteins and the intra-tumoral vasculature never fully re-establishes a normal BBB in brain metastases 7. Both the BBB and BTB have drug efflux transporters expressed in endothelial cells in common and ATP-binding cassette (ABC) transporters can be expressed in some tumor cells 8. Brain tumor capillaries can also overexpress receptors that mediate ligand-dependent drug delivery, which has previously been exploited to enhance drug delivery directly to tumor tissues 9.

### Drug delivery across the blood-brain barrier and blood-tumor barrier

Numerous strategies have now been investigated to either evade or alter the cellular and molecular barriers of both the BBB and BTB. These methods can be sub-categorized into methods of drug modulation, methods of BBB/BTB modulation, mechanical disruption of the BBB/BTB and complete BBB/BTB bypass methods. Strategies to bypass or hijack the BBB are currently being established or optimized. In this review, we discuss all methods currently used in the clinic or under preclinical or clinical investigation, including molecular, biological, and physical methods to overcome the BBB.

## Modulation of drugs to cross the BBB

### Peptide-drug conjugates and modulation of transcytosis

Peptide-drug conjugates are considered as prodrugs due to the covalent coupling of a peptide to a drug via specific linkers. They typically include a cytotoxic agent, a tumor-homing peptide, and a linker between them. Peptide-drug conjugates have been produced by linking drugs with BBB permeable peptides. In recent years peptide-drug conjugates have evolved, with the large-scale production and purification of peptides being simplified while an array of different tumor-targeting peptides have been discovered for different cancer types. This allows for a somewhat personalized therapy to be designed by selecting a tumor-homing peptide and desired physiochemical properties, such as solubility and stability, necessary for conjugation with the therapeutic load.

For a peptide-drug conjugate to be effective, it requires specific features, including a receptor-specific peptide and a stable peptide-drug conjugate with a strong binding affinity for the receptor. The peptide must bind selectively to a specific receptor on the cell surface of the target tissue that is unique or overexpressed in the cancer cells and at sufficient levels to transport the drug into the tumor. The peptide-drug conjugate site and linker must not modify the binding affinity to the target receptor or the stability to ensure that it will reach the tumor site to release the drug, thus limiting off-target toxicity. Some of the most used linear and cyclic peptides are arginine-glycine-aspartic acid, gonadotropin-releasing hormone, somatostatin, epidermal growth factor and Angiopep-2. All these peptides are delivered to cells using endocytosis/adsorptive-mediated transcytosis except for Angiopep-2, which enters the cells via transcytosis via the Low-density lipoprotein receptor-related protein 10 (LRP-1) transporter. These peptides have commonly been paired with cytotoxic agents such as gemcitabine, doxorubicin (Dox), daunorubicin, paclitaxel and camptothecin.

#### Arginine-glycine-aspartic acid (RGD)

The tripeptide arginine-glycine-aspartic acid (RGD) motif is a widely applied peptide carrier and mediates cell attachment by targeting integrins. The RGD motif is contained in various proteins like fibrinogen, fibronectin, prothrombin, tenascin, and other glycoproteins. RGD is recognized by integrins, which play an essential role in cell proliferation, invasion and angiogenesis, and are overexpressed in brain endothelial cells, newly formed vessels and tumor cells 10.

RGD peptide drug conjugates have been used for targeted drug delivery in multiple high grade glioma models 11-16. RGD peptides have been tested in clinical trials (Phase I/II and Phase III) in newly diagnosed, progressive, or recurrent glioma with modest anti-tumor effects observed 17-21.

#### Somatostatin (SST)

Somatostatin (SST) is a neuropeptide produced by neuroendocrine, inflammatory, and immune cells. SST's various physiological functions include working as an endocrine hormone, a paracrine regulator or a neurotransmitter 22. SST is ubiquitously expressed in humans, with high concentrations in the brain, liver, lungs, pancreas, thyroid, gastrointestinal tract, and adrenal gland 22. SST has two active forms: SST-14 and SST-28, are mediated through five distinct GPCR subtypes (somatostatin receptor 1-5; SSTR1-5). SST is thought to be transported across the BBB by both the ATP-powered efflux pump, P-glycoprotein (Pgp), on the plasmatic membranes of the endothelial cells of the BBB and the unidirectional efflux transporter, Multidrug resistance protein 2 (Mrp2), on the apical membrane of brain capillary endothelial cells of the BBB 23.

GBMs express multiple SST receptors, with SSTR1 and SSTR2 being the most frequently expressed in GBM 24. SST analogues linked with Dox (or Dox derivatives) or novel peptides have been successfully used for drug targeting in GBM models 25, 26. These studies demonstrate the efficacy of modifying drugs to target the SSTRs in gliomas for efficient and specific drug targeting of therapeutics however, none of these have progressed to clinical trial.

#### Angiopep-2

Angiopep-2 was derived from low-density lipoprotein receptor-related protein 1 (LRP1) receptor involved in the uptake and processing of amyloid precursor protein in the intracellular compartment inside endosomal vesicles 27. Angiopep-2 has recently attracted attention due to its ability to cross the BBB via receptor-mediated transcytosis after binding to LRP1, making it an appealing drug carrier for brain malignancies. Angiopep-2 has been exploited for transporting cancer therapeutics such as Dox, paclitaxel, camptothecin and etoposide as well as proteins and genetic materials 27-31. Additionally, nanoparticles formulated by Guo and colleagues, statin-loaded Angiopep-2-anchored nanoparticles (S@A-NPs), has been shown to increase LRP1 expression in brain endothelial cells and brain metastatic tumor cells *in vitro*, leading to improved transcytosis. The nanoparticles were also seen to improve median survival in a brain metastatic mouse model when loaded with Dox 32. Other nanoparticle formulations, such as that by Khan and colleagues, are developed to evade clearance by LRP1. They reported that nanoparticles attached to an MMP1-sensitive fusion peptide containing HER2-targeting K and LRP1-targeting angiopep-2 (A), or NPs-K-s-A, showed an increased brain accumulation compared to angiopep-2-decorated NPs in breast cancer brain metastases mouse models 33. ANG1005, consisting of paclitaxel conjugated to Angiopep-2 has been examined in clinical trials to treat brain metastases and high-grade gliomas with early signs of clinical activity 34, 35.

#### Mfsd2a inhibition

Other strategies to modulate transcytosis for improved penetration of the BBB have been studied. Mfsd2a is an essential fatty acid transporter and is a regulator of transcytosis, limiting BBB penetration. Inhibition of Mfsd2a would therefore allow greater permeability. Ju,* et al.* designed tunicamycin-loaded transcytosis-targeting-peptide-decorated-nanoparticles (TM@TTP) loaded with Dox to reversibly inhibit Mfsda2 and enhance transcytosis, showing efficacy in a breast cancer brain metastases mouse model 36. Other nanoparticles developed to improve transcytosis and downregulate tight junction proteins have been investigated, such as the minoxidil-loaded hyaluronic acid-tethered nanoparticles (M@H-NPs), shown to improve delivery of Dox and thereby significantly improving median survival of breast cancer brain metastasis bearing mice 37. A recent, more detailed review on modulation of efflux, modulation of transcytosis and interference of tight junctions has been published by Han in 2021 38.

### Non-covalent avidin-biotin linkage of drugs

The non-covalent interaction of avidin-biotin has been utilized as a nanoscale drug delivery system for pharmaceuticals, including small molecules, proteins, vaccines, monoclonal antibodies, and nucleic acids. Avidin is composed of four identical glycoprotein subunits that bind biotin with high affinity and specificity. The avidin-biotin non-covalent interaction is highly specific and stable against manipulation, proteolytic enzymes, temperature, pH, harsh organic reagents, and denaturing reagents 39. Avidin-biotin technologies have been recently exploited to deliver biologics and chemotherapeutic agents across the BBB via transferrin receptors (TfR).

Avidin-biotin technology is an efficient way to deliver chemotherapeutic drugs to the site of action via monoclonal antibody carriers. TfR monoclonal antibodies function to transport the bound drug into the brain through binding to the BBB TfR. Avidin-biotin technologies have shown promise due to their ability to specifically target and accumulate in tumors, permitting non-BBB permeable drugs to efficiently reach the tumor site, minimizing off-target effects and adverse side-effects. The avidin-biotin delivery system has been used preclinically with paclitaxel (PTX) and with siRNA and clinically in GBM 40-42. Treatment with biotinylated anti-tenascin monoclonal antibody treatment followed by avidin and ^90^Y-biotin showed promising survival rates in a cohort of GBM patients. Survival was 98.4% at 6 months, 79.2% at 12 months, 51.7% at 18 months, and 30.7% at 24 months after treatment 42.

## Nanoparticle drug carriers to cross the BBB

**Figure 3:**
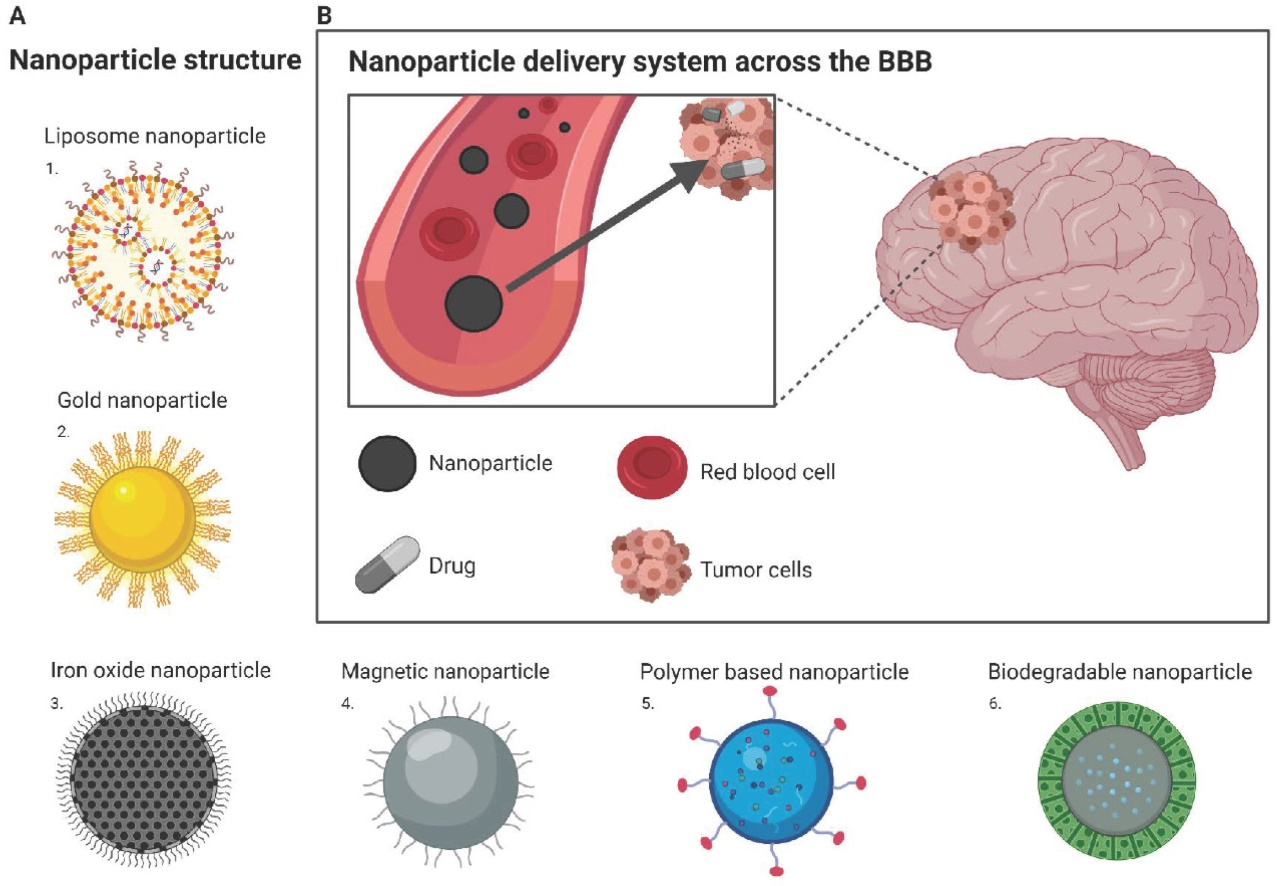
** Nanoparticle drug delivery to the brain (A)** Structure of different nanoparticles: 1. Liposome nanoparticle; 2. Gold nanoparticle; 3. Iron oxide nanoparticle; 4. Magnetic nanoparticle; 5. Polymer based nanoparticle; 6. Biodegradable nanoparticle **(B)** Nanoparticle delivery system across the BBB: Drug is encapsulated within nanoparticle and can freely travel from the blood stream to the tumor site located within the brain. Image was created with BioRender.

Recent breakthroughs and the advancement of nanotechnology offers the potential for patients suffering from CNS diseases a promising route for drug delivery. Nanotechnology is an engineering approach that can be used for medical applications including the development of nanoparticles as drug carriers to enhance delivery through the BBB (Figure 3B). The benefits of delivering nanoparticles include the ability to cross the BBB, increased permeability and retention in cancer cells, and being non-invasive for patients with limited effects on surrounding healthy tissues 43. Nanoparticles used as drug carriers are based on stable elements (inorganic), including gold and iron, or organic nanoparticles, such as liposomes, micelles, and polymer nanoparticles.

### Lipid-based nanoparticles

Lipid-based nanoparticles have either bilayer or multilayers and are capable of transporting both hydrophilic and hydrophobic drugs. Their characteristics increase the stability and solubility of the drugs being delivered across the BBB. In addition, being coated in micelles or polyethylene glycol allows efficient targeting and transportation of the nanoparticles 44, 45. These nanoparticles are stable, an advantage that allows for detailed control of the carrier properties such as size and charge 46. Preclinical studies have investigated lipid-based nanoparticles in combination with various agents, but with limited efficacy in brain cancer.

Liposomes are nanosized, spherical vesicles that are made up of one or more phospholipid bilayers where the polar groups of phospholipids are orientated inside, and there is an outer aqueous phase. They can also contain cholesterol can reduce the permeability of the lipid bilayer and increase liposome stability. Liposomes can be readily encapsulated with a range of drugs within the inside aqueous compartment, the lipid bilayers, and at their interfaces (Figure 3A). Due to the physical characteristics of liposomes, they can incorporate therapeutics that are hydrophilic, lipophilic, or hydrophobic. Hydrophilic agents are encapsulated into the aqueous core or are located between the lipid bilayer interface and the outer water phase of the liposomes. Lipophilic or hydrophobic agents are encapsulated in the hydrophobic core of the lipid bilayers of the liposomes. Liposomes have excellent biocompatibility and biodegradability, drug-targeted delivery, controlled drug release, and low toxicity 47. They can also be modified to improve their blood circulation and BBB permeability by surface functionalization with macromolecules, such as polymers, polysaccharides, peptides, antibodies, or aptamers, on the liposome surface. Further to this, additional properties can also be added to improve delivery according to fluctuations in the target site microenvironment, such as temperature, pH, or externally applied stimuli, such as ultrasound intensity or magnetic fields 48. Specific BBB targeting can occur through harnessing the low-density lipoprotein receptor expressed on brain capillary endothelial cells. Additionally, receptor-mediated endocytosis through conjugating ligands, such as peptides or antibodies (as described earlier), to the liposomes can be employed to interact with specific receptors expressed on endothelial cells. Other approaches include using electrostatic interactions between cationic charged compounds with negatively charged plasma membrane of endothelial cells, allowing for adsorption-mediated transcytosis or endocytosis into the BBB (comprehensively reviewed by Vieira & Gamarra) 48. Currently, liposomal drug delivery is not a readily used technique in a clinical setting for brain tumors.

Clinical results of lipid-based nanoparticles applications used to improve BBB penetration in brain tumor treatment are summarized in Table 1.

### Metal-based nanoparticles

Nanoparticles synthesized with metals such as gold, iron, silver, copper, and others have been studied and are of interest in the use of diagnostic probes in brain tumors. Coatings and functional groups on nanoparticles play a role in improving penetrability while limiting toxicity to surrounding tissues. Variation of the physiochemical properties of nanoparticles, including size, surface coating and charge, shape and chemical composition can significantly alter biocompatibility, distribution, and function, thus making them a potential nano-carrier in neuro-oncology. A study by Guo and colleagues tested varied sizes of different metal-based nanoparticles including cerumin oxide, iron oxide and zinc oxide, along with four different shapes of silver (spherical, disc-like, rod-shaped and nanowires). Results showed that zinc oxide was able to readily cross the *in vitro* BBB model and that spherical and disc-like silver nanoparticles created an easier entry pathway 70.

The use of specific coatings, such as starch, PEG, chitosan, dextran or polysorbate, can enhance penetration, bio compatibility, and systemic half-life by evading the clearance by the reticulo-endothelial system 71.

Gold nanoparticles are a promising agent for brain tumors and have gathered momentum in the advancement of modern nanotechnology (Figure 3A). They can be advantageous in that they can be engineered to target specific tumor markers, encapsulate insoluble drugs, modulate drug release, and improve bioavailability. They were initially designed to carry antineoplastics but are now considered suitable to also carry nucleic acids for gene therapy, inflammatory cytokines, flavonoids, and inhibitors. They are also used as imaging agents, radiosensitizers by inducing BBB disruption and theranostic agents.

Iron oxide nanoparticles typically have a solid iron oxide core with a polymer coating (Figure 3A). They are unique and have been shown to have on-target effects due to their magnetic guidance and ligand targeting 45. External magnetic fields can also be applied to metal-based nanoparticles to direct the nanoparticles loaded with therapeutics into the desired area of the brain (Figure 3A). Additionally, the radiofrequency field allows for magnetic nanoparticles to exert heat and temporarily open the BBB in a localized area 72. Magnetic nanoparticles typically have an iron oxide core, mainly magnetite Fe_3_O_4_, and are divided into paramagnetic nanoparticles (PMNPs) that are 100 nm in diameter and superparamagnetic iron oxide nanoparticles (SPIONs) that are less than 100 nm. SPIONs are favorable due to their small size, susceptibility to magnetism, prolonged circulation and retention in tissues, and low toxicity 71, 73. However, not much is known about iron's potential side effects and toxicities on genetic material, nervous system functioning, and development. Careful consideration of surface coating materials and size are paramount in developing a nanoparticle and are major determinants of effects on cellular structures as well as toxicities.

Preclinical and clinical studies using metal-based nanoparticles and their functionalizations for the treatment of brain tumors are summarized in Table 2.

### Polymer-based nanoparticles

Polymer-based nanoparticles are a form of organic nanotechnology produced by polymerizing various monomers and loaded with the desired agent (Figure 3A). The characteristics of polymeric nanoparticles are also tunable such as charge, size, shape, and surface ligands, to suit specific applications, thus targeting the BBB. Synthetic polymers, such as poly (alkyl cyanoacrylate), poly(lactic-co-glycolic acid), poly-*ε*‐caprolactone, and polyamidoamine dendrimers have been developed but are not without limitations. Drawbacks include low biodegradation rate due to hydrophobicity, toxicity, high cost, and purity 90. Although synthetic polymeric nanoparticles have been investigated and developed over time, clinical success is still limited. Hence further study has been focused on the kinetics of drug loading, targeting, and release. Natural polymeric nanoparticles have also been investigated and seem to solve the issues that synthetic polymers have, such as being less toxic, more sustainable, biodegradable and less costly 90. Naturally occurring polymers include, but not limited to, chitosan, a commonly used polysaccharide, and alginate, a polysaccharide from brown seaweed.

Polymeric nanoparticles for CNS delivery are still under investigation, with some showing promise in the preclinical setting. Other studies show that nanoparticles, combined with mechanical BBB disruption methods such as focused ultrasound and convection-enhanced delivery, are more beneficial 91. A detailed review on this area has been published recently by Zhang and colleagues 90.

A summary of preclinical studies for polymer-based nanoparticle delivery for brain tumor treatment is listed below in Table 3.

## Blood-brain barrier modulation

### Osmotic opening

Hyperosmolar BBB disruption is the shrinkage of endothelial cells caused by hyperosmolar plasma conditions, which cause the vasodilation of capillaries and opening of tight junctions 100. This loss of water from brain tissue into the circulation is caused by a higher osmotic pressure, where this calcium-mediated process causes changes in the endothelial cytoskeleton and junctional proteins, modifying the junctional width 100. The influx of water into the brain assists in drawing chemotherapeutic drugs in, thereby enhancing therapeutic efficacy. In 2004, Brown *et al.* discovered a regional variability in mannitol BBB opening effectiveness, where sucrose permeability post mannitol infusion was higher in cortex and midbrain compared to cerebellum and brainstem 100. In a preclinical rat model, infusion of hyperosmolar compound (1.6M mannitol) was tested, and permeability was analysed by ^86^Rb^+^ (a marker for K+ transport), ^14^C sucrose and Evans blue albumin. Only sucrose and Rb had increased permeability, and sucrose showed regional differences - cortex and midbrain higher permeability than brainstem and cerebellum 100.

To date, mannitol has shown promising results in PCNSL. Intraarterial hyperosmolar mannitol infusion is considered safe and has been used to transiently open the BBB to increase the penetration of macromolecule chemotherapies by 100-fold and thereby improve the response to treatment 101. A safety and efficacy multicentre study on PCNSL showed that 40 out of 43 patients had a complete response to intra-arterial Methotrexate (MTX) before hypertonic mannitol in combination with IV cyclophosphamide and etoposide 102. This same study also investigated neuroectodermal tumors, germ cell tumors and GBM treated with intraarterial carboplatin after hypertonic mannitol with IV cyclophosphamide 10 min before mannitol, as well as IV etoposide for several days, which resulted in patients with stable disease and minimal complications 102. A long term follow up study in 2000, in patients with PCNSL treated with MTX and mannitol BBB disruption without radiation therapy, showed 36 out of 48 patients exhibited complete response with a 5-year survival rate of 42% and median survival of 40 months and no cognitive loss 103. This demonstrates the safety and feasibility of this approach, but Phase 3 trials are needed to determine whether the addition of mannitol improved outcomes.

BBB disruption with hypertonic mannitol is reversible with minimal permanent brain changes. The maximum osmotic effect on the BBB lasts for 10 min but can be enhanced by Na^+^/Ca^2+^ channel blockers for 30 min. This is beneficial as the brains receive longer exposure to the drug and less systemic exposure. A prolonged opening may also be beneficial for drugs that are only active in specific phases of the tumor cell cycle, such as MTX. However, limitations for this approach include repeated hospitalisation, associated with a need for sedation, and toxicities include neurological deficits, strokes and seizures 104.

Despite being developed in the 1970s, mannitol is still being explored to help improve chemotherapy penetration to brain tumors. A Phase I/II clinical trial (NCT00303849) is currently recruiting patients to undergo mannitol BBB disruption with chemotherapies carboplatin, melphalan, etoposide, and sodium thiosulfate for the treatment of previously treated brain tumors. In addition, intra-arterial infusion of cetuximab is being investigated with mannitol BBB disruption for newly diagnosed GBM (NCT02861898).

There is currently a lack of completed studies using mannitol to treat other primary brain tumors other than GBM. The safety and feasibility demonstrated in PCNSL suggests that application of mannitol warrants further research.

### Inhibiting drug efflux

ATP-binding cassette (ABC) transporters are a superfamily of integral membrane proteins that transport solutes across cellular membranes by ATP. Pgp, MRP and breast cancer resistance protein (BCRP) are ABC transporters expressed in the BBB and play a role in limiting the entry of therapeutics into the brain and creating multi-drug resistance. This poses a significant problem in delivering therapeutics into the brain, especially in brain tumor treatment.

Tournier, *et al.* showed that ABCB1 (Pgp) and ABCG2 (BCRP) efflux transporters at the BBB and work concurrently to restrict access to tyrosine kinase inhibitors. Elacridar is a well-known ABCB1 and ABCG2 inhibitor and has been studied in CNS metastases models. In mice, elacridar successfully increased brain uptake of erlotinib, however, preclinical data in humans did not show the same success 105. Elacridar however has undergone significant preclinical and clinical study and has been shown to significantly increase brain penetration of dasatinib, gefitinib, and sorafenib 106-108. Novel molecules have been investigated and continue to be developed to provide potent Pgp inhibition. A recent study by Fallacara and colleagues developed a dual Src/Pgp inhibitor, Si306, and found in *in vitro* studies that it inhibited cell growth 2-fold that of dasatinib and had dose-dependent suppression of Pgp activity 109. They also found that administration of the prodrug form of Si306 could increase median survival in mice harboring orthotopic GBM tumors 110.

Drugs have also been modified to have a lower affinity for efflux transporters. A study by Becker, *et al.* investigated PI3K/mTOR inhibitors for the treatment of GBM in mouse models where they modified GDC-0980, an inhibitor with high affinity for efflux transporters, and its analog GNE-317. Both inhibitors displayed decreased efflux and 3-fold higher drug penetration into the tumor core, as well as decreased histological staining for effector proteins 111.

Currently, research of efflux transporter inhibition is still progressing toward finding potent dual ABCB1/ABCG2 inhibitors. A study by Strope, *et al.* in 2019 investigated a more specific ABCG2 inhibitor, botryllamide G, and found that it could significantly increase brain concentrations of lapatinib. However, ABCB1 would also need to be blocked for a complete efflux transporter inhibition 112. In addition, off-target effects of Pgp inhibition are a significant pitfall since ABCB1 and ABCG2 are expressed in the gut, liver, and kidney.

A phase 1 clinical trial with tariquidar (XR9576) for inhibition of Pgp, in combination with Dox, vinorelbine or docetaxel was conducted in pediatric patients with a range of solid tumors, including brain tumors (NCT00011414) 113. It found that the Pgp substrate fluorescent dye ^99m^Tc-sestamibi had increased tumor accumulation by 22% with administration of tariquidar. Out of 29 enrolled subjects, one patient had a complete objective response, and 2 had partial responses. Toxicities from tariquidar were minimal, including transient hypotension, loss of taste and nausea. However, when administered in combination with chemotherapeutic agents' docetaxel and vinorelbine, systemic clearance was reduced thereby increasing drug exposure causing drug associated toxicities 113.

Thus, the large availability of preclinical studies into Pgp inhibitors has not translated well into a clinical setting, and the search for more highly potent, selective, and efficacious Pgp inhibitors is still ongoing.

### Chemical modification of blood-brain barrier and blood-tumor barrier using TNFα

Inflammatory cytokine tumor necrosis factor α (TNFα) has been shown to disrupt the BBB by decreasing levels of tight junction proteins, causing changes in endothelial cell adhesion, and compromising barrier function and integrity 114. Human TNF conjugated with a cyclic peptide Cys-Asn-Gly-Arg-Cys NGR (NGR-hTNF) targets CD13+ vessels has been tested at low doses and has been found to increase vascular permeability and assist in drug penetration in central nervous system (CNS) lymphoma and melanoma in animal models 114, 115. NGR-hTNF is also in phase II and III clinical trials for different tumor types with and without chemotherapy and has shown safety and activity. A high-dose NGR-hTNF phase I trial was completed and showed that the dose could be safely escalated while maintaining tolerability with chills being the only toxicity observed 116. R-CHOP is a commonly used chemotherapy for primary CNS lymphoma (PCNSL) comprising rituximab, cyclophosphamide, doxorubicin hydrochloride, vincristine, and prednisolone and has been tested in combination with NGR-hTNF. In a clinical trial with 12 PCNSL patients, 9 displayed rapid tumor regression after therapy, with a complete response in 8 patients (NCT03536039) 114. There were 2 severe adverse events and grade 4 toxicities that arose in this study, but both were resolved and treated. While it is not possible to evaluate the clinical efficacy of TNF-α with this small number of patients, the response rate and lack of toxicity is encouraging. This is the only clinical application of TNF-α for the disruption of the BBB that has been studied to date.

### Mechanical disruption of the Blood-brain barrier/Blood-tumor barrier

#### Focused ultrasound

Focused ultrasound (FUS) can focus acoustic energy into a focal spot to deliver selective BBB disruption and enhanced permeability 117. FUS can provide reversible BBB disruption to enhance permeability by concentrating acoustic energy to a focal point. FUS can be used in conjunction with clinically available drugs and is a relatively benign procedure that can be modified to match chemoschedule. Microbubbles have been integrated into the FUS approach, which has helped focus the effect of FUS to the blood vessel walls, thereby minimizing the damage to the surrounding healthy brain tissues. In MB-facilitated FUS, circulating microbubbles interact closely with the low-intensity FUS, causing a temporary disassembly of tight junctions and increased permeability of the BBB 118. Research has shown that repeated application of FUS and microbubbles to open the BBB over a long term (4-20 months) did not result in oedema in the majority of the cases 119. There was however, a significant increase of reaction time during a neurotoxicity test task on the day of FUS and microbubble application which returned to baseline within 4-5 days, demonstrating the safety of this method 119.

Microbubbles and FUS have been successfully combined with multiple drugs, including Dox, TMZ, BCNU (1,3-bis(2- chloroethyl)-1-nitrosourea) bevacizumab and MTX in preclinical models 120-126. It has even evolved further to be used with macro-agents such as magnetic nanoparticles, small interfering siRNA, and even stem cells 127-130. In addition, focused ultrasound is currently in clinical trials for brain tumors, including low-grade glioma and GBM, as well as in Alzheimer's Disease, Parkinson's disease dementia and breast cancer with brain metastasis 131, 132. These studies provide preliminary evidence that FUS can transiently increase BBB permeability and enhance local concentrations of antitumor drugs to further inhibit tumor development. One of the main limitations of this technology is that FUS is usually only targeted to small brain volumes or areas and therefore only small regions of the BBB are opened.

#### Stereotactic radiation therapy

Radiation therapy plays a prominent role in treating many types of cancer, with almost 50% of patients receiving ionizing radiation at part of their treatment 133. There are different types of radiation treatments that are used based on tumor type, location and the radiosensitivity of the cancer cells. Radiation therapy works by depositing its energy in the tumor site to damage the DNA of the tumor cells thereby inducing apoptosis of the cells. Unfortunately, due to the nature of ionizing radiation, it can also cause DNA damage to surrounding tissues, thereby damaging glial, neuronal, and vascular cells of the brain, which is often considered an adverse consequence of ionizing radiation. However, because both endothelial cells and oligodendrocytes are radiation responsive, ionizing radiation can be used in a targeted and controlled manner to purposefully damage tissue and increase the BBB permeability 134.

Previous studies have shown at both the *in vitro* and *in vivo* level that high doses of radiation have been shown to induce BBB permeability, tight junction morphology changes, reductions in cell density, and the formation of actin stress fibers in cerebral endothelial cells in healthy regions of the brain 135, 136. Although these studies show promise, optimal dosing remains undetermined (with BBB permeability seen at doses ranging from 0.1 Gy to 20 Gy), the therapeutic window remains undetermined and the side effects associated with this method, including oedema, remain concerning. Radiation therapy has gained interest with its potential to be combined with nanoparticle technology for targeted therapy delivery to the tumor site and immunotherapy 137. However, very few clinical trials have been completed using this method (NCT00019578) with some still in the active or recruiting phase (NCT03561896, NCT02974803).

#### Electric field modulation

Electric field modulation has been used in other fields of medicine and has recently been used as a novel treatment method in oncology. Electric field modulation has recently been re-termed tumor-treating fields and has been used to open the BBB for chemotherapeutic drug delivery in a method known as electrochemotherapy 138. Tumor-treating fields therapy delivers low‐intensity (1-3 V/cm), intermediate‐frequency (100-300 kHz), alternating electric fields to the tumor using transducer arrays placed on the skin around the region of the body containing the tumor 138. This method causes irreversible electroporation through the needle electrode delivery of electric pulses inducing nonthermal focal ablation 139. This causes cell death due disrupted membrane integrity.

Preclinically tumor treating fields have been used successfully to inhibit cell proliferation in cancer cell lines, including human glioma, non-small-cell lung carcinoma, breast carcinoma as well as animal models of glioma and melanoma and in combination with chemotherapeutics 140-142. There are currently numerous clinical trials in the recruiting phase testing tumor treating fields for the treatment of brain metastases in small cell lung cancer, and ependymoma. Clinical trials on brain metastasis from primary lung cancer, brain cancers and GBM have been completed with some evidence that tumor-treating fields can increase the permeability of the BBB 143, 144. Unfortunately, clinical effects have been small and the practicality of applying electric field modulation is burdensome with devices needing to be worn for 20 h a day.

#### Laser induced thermotherapy

Laser induced thermotherapy (also known as interstitial laser thermotherapy) (LITT) was first described in 1990 by Kiesslin, *et al.* who introduced the hypothesis that laser light could be used to cause a concentrated disruption by focally applying a neodymium-doped yttrium aluminium garnet (Nd:YAG) laser pulse 145. The procedure involves the use of a stereotactic device to introduce optical fibers to deliver laser light interstitially. This laser light is deposited at low power and over a long exposure time to increase the target area temperature. When the thermal threshold temperature between 50 °C and 80 °C is reached, protein denaturation and irreversible tissue coagulation occurs resulting in permanent tissue damage 89. Lower temperatures of 43 °C to 45 °C for more than 10 min will sensitize cancer cells to chemotherapy and radiation therapy. Laser therapy has been used in combination with tumor-localizing photosensitizers such as prodrug 5-aminolevulinic acid (5-ALA). 5-ALA has been employed for the treatment of glioma due to its tumor specificity and rapid systemic clearance. 5-ALA-laser combination treatment disrupts the BBB rapidly via developing and enlarging of endothelial gaps. LITT, much like FUS, can be used in conjunction with nanotechnologies to target drug delivery. In 2011, Choi, *et al.* proved that near-infrared ultrashort pulsed laser in combination with large molecules such as nanoparticles and genetically engineered viruses, could penetrate the BBB 146. LITT has since been shown preclinically to enhance survival in a GBM model 147.

Unfortunately, at the clinical trial level there is little to no reported information on LITT in brain cancers even though some clinical trials have run to completion (NCT01651078, NCT02451215, NCT00207350, NCT01851733) or are currently still in the recruitment phase (NCT00787982, NCT02389855, NCT02392078, NCT02311582, NCT04181684) 148, 149. Clinical trials showed that LITT may have a role in the management of radiation necrosis and can allow a timeframe for additional therapeutic intervention such as further local radiation or systemic therapies 150.

## Approaches that bypass BBB/BTB

### Intrathecal and intraventricular administration

Intrathecal administration is the delivery of therapeutics directly into the cerebrospinal fluid (CSF) that surrounds the spinal cord, termed the intrathecal space or subarachnoid space, bypassing the BBB and achieving higher therapeutic concentrations due to the proximity to the target, thereby limiting potential systemic toxicities 151. There may also be lower levels of enzymatic breakdown of drugs in the CSF compared to plasma which can lead to an increase in target drug concentrations 151. Intrathecal administration was firstly used for the delivery of analgesics in the 1980s for acute and chronic pain but has been further developed for use with chemotherapeutics or other biological macromolecules.

Intrathecal therapeutics can be administered by an external pump or implantable devices. Implantable infusion pumps, however, are costly, invasive and cause secondary issues such as formation of granulomas and infection which may lead to catheter dysfunctions, compression of the spinal cord and paralysis 152. Single injection strategies are favored over implantable devices or repeated intrathecal administrations but need to have robust stability to reside in tissues for therapeutic effect and combat the physiological characteristic of bulk flow 151. Administered molecules must also be stabilized and have good penetrative properties to infiltrate the parenchyma 151. However, there are limitations that have hindered this technique from further development. Discrepancies between CSF concentration and parenchymal concentrations suggest a physiological CSF-brain barrier that could limit delivery into brain tissue, along with high rates of clearance from CSF 151.

Various *in vivo* studies have been conducted using intrathecal delivered agents for leptomeningeal dissemination. A study using intrathecal MX2, an anthracycline inhibiting topoisomerase II, in a rat model of dissemination showed only a modest increase in survival, which could be explained by inadequate infiltration into parenchyma 153. A patient study investigating intrathecal triothriethylenephosphoramide (thio-TEPA) for ependymal or leptomeningeal dissemination from anaplastic astrocytoma or GBM was conducted and found a modest improvement in survival at 10 months 154.

Intraventricular administration is the placement of a subcutaneous device, an Ommaya reservoir, with a catheter in the lateral ventricle, allowing therapeutics to enter directly into the ventricular CSF. The benefit of this technique is allowing for a more consistent drug distribution with higher concentrations achieved in comparison to intralumbar injections 155. This technique is regularly used in pediatric tumors, such as AT/RT and infant medulloblastoma with conventional cytotoxics, however it is used in this setting for the treatment or prevention of leptomeningeal spread rather than intraparenchymal disease.

### Intranasal delivery

Intranasal delivery has been investigated in the context of neurological diseases such as neurodegenerative conditions, migraines, schizophrenia, etc 155. The intranasal route can bypass the BBB via the olfactory and trigeminal nerve pathways. This method is safe and non-invasive with an increased rate of absorption and capable of achieving higher concentrations due to the high vascularization of the nasal epithelium 156. The use of intranasal delivery is limited for brain tumors, but early data is promising. Studies in rats show accumulation of MTX, raltitrexed and 5-fluorouracil in the brain after intranasal administration 157-159. The intranasal delivery of GRN163, a telomerase inhibitor, in a human GBM xenograft in rats showed good uptake and distribution in the tumor 4 h after delivery and a significant increase in survival at median 75.5 days compared to 35 days in control groups 160. Intranasal administration of natural compounds, curcumin and anthranoid 4,5-dihydroxyanthraquinone-2-carboxylic acid or rhein, have been shown to be efficacious in GBM mouse models by exhibiting tumor regression and extensions in survival 161, 162. MTX, commonly used for a wide range of cancers but a poor penetrator of the BBB, was studied for intranasal delivery in rats. It was found that CSF concentration of MTX 15 min after intranasal delivery was higher than in plasma, indicating rapid absorption and transport into CSF 163. Chitosan microspheres encapsulated MTX were shown to be more effective than MTX solution and intravenous MTX by higher concentrations of MTX found in rat brain tissue 164. TMZ, despite its good BBB penetrability, is ineffective in prolonging survival in patients with GBM. A study comparing oral, intravenous or intranasal administration routes of TMZ in a rat glioma model showed that intranasal delivery was the most effective at reducing tumor volume and significantly increasing survival 165.

Perillyl alcohol, a natural compound, was investigated via the intranasal route for the treatment of GBM where 37 recurrent glioma patients were enrolled and treated with the agent by inhalation and resulted in 14 patients showing either partial response or stable disease 166. Subsequently, a larger cohort study consisting of 198 patients with primary GBM, grade III astrocytoma or anaplastic oligodendroglioma showed intranasal perillyl alcohol could significantly prolong survival compared to control group, with 19% of patients under remission after 4 years 167. Another study with 198 recurrent GBM patients treated with perillyl alochol inhalation was conducted over 4 years and showed significant survival increase as well as 19% of the cohort showing clinical remission over 4 years 168. Following these promising results, a safety and efficacy study for perillyl alcohol in recurrent grade IV glioma was initiated in 2016 and is currently undergoing recruitment (NCT02704858).

However, limitations of the intranasal technique includes small volumes of administration, limited retention in the nasal cavity for absorption, potential mucociliary clearance or enzymatic drug degradation 169. Despite the downfalls of this administration route, studies have been conducted and shown to be successful in the treatment of brain tumors and is currently still under investigation.

### Intratumoral and intracavitary delivery

Intracavitary chemotherapy is administered in the form of implantable biodegradable polymer wafers in the brain or in a tumor cavity during surgery, allowing for a continuous release of chemotherapeutics locally at the tumor site and limiting systemic exposure. Direct injection of chemotherapeutics was the earliest method for intracavitary delivery, by injecting directly into the resection cavity, surrounding parenchyma or the ventricle, using a needle or implantable catheter with reservoir. This method has been used for various therapeutics such as chemotherapies, radioactive compounds, and antibodies 170. Despite the relative simplicity of this approach, intracavitary or intraparenchymal injections involves surgical intervention, increasing the risk of complications. Additionally, this technique requires an optimal concentration gradient and distribution which is difficult to achieve and could result in toxic concentrations in the surrounding areas 170.

Bortezomib, a proteasome inhibitor, is ineffective for the treatment of GBM, however implantation of a mini-osmotic pump to intratumorally deliver the drug was shown to be successful and significantly increased survival in an orthotopic GBM mouse model 171.

To overcome systemic toxicity of chemotherapies, Gliadel® wafer was developed, which is composed of polyanhydride polymer impregnated with BCNU for the treatment of malignant brain tumors. Development of a BCNU implantable polymer that releases the drug directly into the CNS has made it possible to achieve high local drug concentrations while minimizing systemic toxicity and circumventing the need for a drug to cross the BBB. BCNU has been successfully used preclinically and has since shown clinical success in patients with primary malignant gliomas. However, some adverse side effects have been reported, such as increased cerebrospinal fluid leaks and intracranial hypertension 172. More recently, Gliadel® has been used in combination with TMZ and carmustine preclinically to treat GBM demonstrating a significant survival benefit *in vivo* 173, 174. Gliadel wafers are Food and Drug Administration (FDA) approved and used in adult GBM. A pediatric study with Gliadel wafers combined with low-dose etoposide for recurrent anaplastic ependymoma but was found to be ineffective as relapse occurred 4 months after implantation 175. Often, Gliadel wafers are used in conjunction with other therapeutics to improve efficacy. In a Phase II trial (NCT00362921), Gliadel was combined with O6-benzylguanine infusions in recurrent GBM patients. The overall survival of the fifty-two patients enrolled was 82%, with 1-year and 2-year overall survival of only 47% and 10%, respectively. Toxicities documented were hydrocephalus, CSF leak and infection 176.

Another biodegradable polymer, n-butylidenephthalide, suppressed growth of subcutaneous rat and human brain tumors, reduced GBM tumor volumes and significantly prolonged the survival rate in a preclinical model and has since been incorporated into the 'Cerebraca wafer', which is the first human-use drug product and is a biodegradable implant comprised of (Z)-n-butylidenephthalide ((Z)-BP) and Carboxyphenoxypropane-Sebacic Acid Copolymer (NCT03234595) 177.

A summary of the preclinical investigations and clinical trials for the use of biodegradable polymer wafers are summarized in Table 4.

Intratumoral delivery can be a carried out in a range of techniques, such as local injection, surgical insertion of a catheter with a reservoir, or convection enhanced delivery (CED). Intratumoral delivery of bleomycin in a phase 1 clinical trial was done via an Ommaya reservoir, an implantable device with a delivery tube directed to the center of the tumor and used to treat patients with recurrent GBM. The maximum tolerated dose and recommended Phase II dose was determined and no dose limiting systemic toxicity was documented 178. Intratumoral delivery of DNX-2401, an oncolytic adenovirus, was investigated in a phase 1 clinical trial (NCT00805376), where recurrent malignant glioma patients received the virus by either a single intratumoral injection or single intratumoral injection followed by surgical resection and further virus injection into the cavity via an implanted catheter. The 3-year overall survival of the first group was 20% and 3 out of 25 patients exhibited a 95% decrease is tumor size 21. Various other viruses are currently in clinical investigation for high-grade gliomas, such as Delta 24 RGD (NCT00805376, NCT01582516), PVSRIPO with lomustine (NCT02986178), Reovirus with sargramostim (NCT02444546), and seems to be a promising avenue utilizing intratumoral delivery.

Monoclonal antibodies may be used as vectors for delivery of local adjuvant radiotherapy. A phase II study by Reardon, *et al.* in 2002 detailed the use of ^131^I-labeled antitenascin monoclonal antibody 81C6 in post-surgical resection cavities in 33 patients with malignant gliomas. Median survival was greatly increased to 86.7% compared to historical controls, and 79.4 weeks for specifically GBM patients. At 93 weeks follow up, 11 patients survived. Side effects included treatment-related hematologic toxicity that was treatable in 9 patients, neurologic toxicity in 5 patients, and radionecrosis in 1 patient 179. Intratumoral and intracavitary based delivery are emerging as a valuable technique for delivering viral vectors, antibodies, and radiation. Although promising, none have been approved by the Food and Drug Administration.

### Convection enhanced delivery

Convection enhanced delivery (CED) is a drug delivery technique developed in the early 1990s. This method relies on a continual hydraulic pressure to distribute a homogenous supply of any sized therapeutic molecule directly the site. Although this technique demonstrates a direct route for administration, this method is highly invasive as a catheter is inserted through burr holes into the interstitial spaces of the brain 181. Careful placement of the catheter in the interstitial space of the brain allows molecules, independent of size to enter, thus allowing a wide range of cancer therapeutics to reach the brain. *In vivo* studies were conducted in DIPG and pediatric HGG mouse models, where liposomal Dox was delivered by CED into the pons or the thalamus. However, excessive toxicity was seen with injection of Dox into the pons, indicating a potential issue with using CED in certain anatomical locations 182. Efforts have been focused on the use of recombinant toxins delivered by CED for the treatment of brain tumors, where a variety have reached the stage of clinical testing. An early phase II trial showed the use of transferrin-CRM107 in malignant gliomas, which resulted in 35% of the patients having complete or partial responses, however some toxicity to normal tissue was observed, as well as increased cerebral edema, impeding further investigation 183. CED delivery of interleukin-13 with bacterial toxin PE38QQR showed promise in early phase clinical trials for malignant gliomas (NCT00024570, NCT00024557). The largest multicenter Phase III trial for CED was the PRECISE trial, which was commenced for patients with recurrent GBM. Significant improvements in median survival and progression-free survival were seen with CED of IL13-PE38QQR when compared to a Gliadel control arm (NCT00076986) 184. This agent has also been explored in DIPG, where a Phase I trial in 2018 recruited 5 DIPG patients for CED delivery of IL13-PE38QQR, which resulted in temporary cessation of disease progression in 2 of the patients. However, all 5 patients exhibited progression by 12 weeks after first infusion, and 2 patients reported side effects of cranial nerve deficits and lethargy after infusion (NCT0088061) 185.

Chemotherapy drugs with low BBB penetrability, such as topotecan and PTX, have been investigated with CED delivery in patients with malignant gliomas, showing tumor regression in 69% of patients and 73% response rate, respectively. However, side effects were observed: dose-dependent meningitis and infusate leakage into the CSF 186, 187.

Clinical investigation of CED for the delivery of topotecan into two patients harboring DIPG tumors, has demonstrated feasibility and safety of the technique at low infusions rates, however no prolongation of survival was observed (NCT00324844) 188.

Several Phase I clinical trials are still underway for an investigation into CED for malignant gliomas and DIPG. A more in-depth review by Mehta, *et al.* was published in 2017 that details ongoing clinical trials 181.

Anatomical differences still pose a major problem for implementing CED. Brainstem tumors such as DIPG involve delicate structures that are sensitive to invasive procedures. Further developments into improving catheter design as well as precise placement to prevent reflux and enhance distribution are currently being investigated. Additionally, imaging should also be used during CED infusions to monitor drug distribution and retention.

## Conclusion

The BBB is a unique and complex structure that is a significant impediment to effective drug delivery to the brain for the treatment of brain tumors. This review detailed the established and emerging techniques investigated to bypass the BBB, from modulation of drugs to modulation or mechanical disruption of the BBB. The future directions of improving drug penetrability into the brain for treating brain tumors greatly rely on technological advancements. Furthermore, clinical trials for adults and children are pivotal for the further development of these techniques. The discovery of a safe and efficacious approach to bypassing the BBB for clinical use for the treatment of brain tumors is urgently needed and has the potential to change the course of brain tumor therapy greatly.

## Figures and Tables

**Table 1:** Liposomal drug delivery in brain tumors

Liposomal Drug Delivery	Nanoparticle Size	Functionalization	Drug	Nanoparticle Status	*In vivo* results	Clinical Use	Clinical Trial	Outcomes
Lipid-based nanoparticles	1-500nm	LBNP conjugate	Epirubicin 49, 50, Cisplatin 51-54, Bleomycin 55, 56, CPT-11 57, edelfosine 58, TMZ 59, 60, vincristine 59, 61	LBNP CPT-11 (Phase I)	Accumulation of edelfosine in xenograft glioma mouse model 58, TMZ increased cytotoxic effect and higher antitumor efficacy 60	GBM, Gliosarcoma, Anaplastic astrocytoma, Anaplastic oligodendroglioma, HGG	NCT00734682 (completed), NCT02022644 (active, not recruiting)	No unexpected toxicities when given LBNP CPT-11 via IV
		wheat germ agglutinin 62	Daunorubicin 63-66	Investigational compound in pre-clinical studies	Wheat germ agglutinin daunorubicin demonstrated strong capabilities in crossing the BBB in glioma bearing mice 62			Not yet reported
		non-PEGylated LBNPs (Myocet®) 67	DOX 67, 68	Myocet® (Phase I)	Treatment of Myocet® showed higher concentration of DOX in brain and spleen, lower concentration of DOX in heart compared to DOX alone 68	Recurrent/refractory HGG, malignant glioma	NCT02861222 (completed)	Myocet® demonstrated acceptable safety
		positive-charged LBNPs 69	PTX 69	Investigational compound in pre-clinical studies				Not yet reported

Abbreviations: LBNPs: lipid-based nanoparticles; PEGylation: polyethylene glycol; PTX: paclitaxel; DOX: doxorubicin; TMZ: temozolomide; CPT-11: irinotecan; GBM: glioblastoma; HGG: high-grade glioma; IV: intravenous; Myocet®: non-polyethylene glycol liposomal doxorubicin encapsulation.

**Table 2:** Metal-based nanoparticle drug delivery in brain tumors

Metal Drug Delivery	Nanoparticle Size	Functionalization	Drug	Nanoparticle Status	*In vivo* results	Clinical Use	Clinical Trial	Outcomes
Gold nanoparticles	5-400 nm	AuNP conjugate	Cisplatin 74, DOX 75, 76, L-aspartate TMZ 77	Investigational compound in pre-clinical studies	Efficient BBB permeability of AuNP DOX and PEG TAT Gd(3+) 75			Not yet reported
		PEG TAT 75	DOX 75, Gd(3+) 75					
		gellan gum 78	anthracycline ring antibiotic DOX hydrochloride 78	Investigational compound in pre-clinical studies	Further investigation needed for *in vivo* experiments 78			Not yet reported
		sperhical nucleic acid (SNA) 79	cytotoxic agents	SNA NU-0129 (Early Phase I)	SNA penetrated BBB and BTB in GBM mouse model 79	Recurrent GBM, Gliosarcoma	NCT03020017 (completed)	NU-0129 well tolerated in GBM, patients had no unexpected adverse effects, showed initial evidence of crossing the BBB
Iron oxide nanoparticles	1-100 nm	DOX-EDT-IONPs 80	DOX 80	Investigational compound in pre-clinical studies	DOX-EDT-IONPs increased blood penetration of these nanoparticles 80			Not yet reported
		IONP conjugate	Chlorotoxin 81, 82, gemcitabine 82, PTX 83, 5-fluorouracil 84	Investigational compound in pre-clinical studies	Efficient chlorotoxin and gemcitabine release activated by high-frequency magnetic field in GBM murine model, successful cellular transport 85			Not yet reported
Magnetic nanoparticles	10-100 nm	magnetic nanoparticle conjugate	magnetic PTX 86	Investigational compound in pre-clinical studies	Efficient BBB permeability of magnetic PTX following IV injection, survival prolonged using magnetic PTX in glioma-bearing rats 86			Not yet reported
		PEG, chitosan, dextran, polysorbate 71, 87, collagen, glycine, glutamine acid 88	cytotoxic agents	Investigational compound in pre-clinical studies	Successful cellular transport, pronounced cytotoxic action of magnetic nanoparticle therapeutics in brain astrocytoma-bearing mice 89			Not yet reported

Abbreviations: AuNPs: gold nanoparticles; TAT: transactivator of transcription; SNA: spherical nucleic acid; DOX: doxorubicin; TMZ: temozolomide; GBM: glioblastoma; PTX: paclitaxel; PEG: polyethylene glycol; DOX-EDT-IONPs: DOX-chlorotoxin stabilized with trimethyoxysiylpropyl-ethylenediamine triacetic acid; Gd(3+): gadolinium; IV: intravenous; BBB: blood brain barrier; BTB: blood tumor barrier; IV: intravenous.

**Table 3:** Polymer drug delivery preclinical studies in brain tumors

Polymer Drug Delivery	Nanoparticle Size	Functionalization	Drug	Nanoparticle Status	*In vivo* results	Outcomes
Polymer-based nanoparticles	1-1000 nm	PLGA 92	Docetaxel 92, PTX 93,	Investigational compound in pre-clinical studies	PLGA docetaxel injected peritoneally were able to penetrate the BBB in C57BL/6 mice 92	Not yet reported
		PBCA 94	antibiotics, cytostatics, DOX 95, CNS-active drugs 94	Investigational compound in pre-clinical studies	PBCA DOX produced high antitumor effect against intracranial GBM in rats 95	Not yet reported
		PBAE 96	anticancer plasmid DNA	Investigational compound in pre-clinical studies	PBAE anticancer plasmid DNA were able to penetrate orthotopic brain tumor tissue in rats 96	Not yet reported
		polysorbate 80-coated PBCA 95, 97, 98	DOX^95^, hexapeptide dalargin 97	Investigational compound in pre-clinical studies	Increased accumulation of NPs in intracranial glioma tumor-bearing mice 99	Not yet reported

Abbreviations: PLGA: PEGylated-poly (L-lactic co-glycolic acid); PBCA: polybutylcyanoacrylate; PBAE: poly(β-amino ester); DOX: doxorubicin; PTX: paclitaxel; CNS: central nervous system; GBM: glioblastoma; DNA: deoxyribonucleic acid.

**Table 4:** Biodegradable polymer wafers for the treatment of brain tumors

Biodegradable polymers	Polymer type	Drug	Status	Clinical Use	Clinical Trial	Outcomes
150-190nm	PLGA wafer	5-fluorouracil	Investigational compound in pre-clinical studies			Not yet reported
	Gliadel® wafer	Carmustine with 5-ALA followed by RT and TMZ	Phase II (completed)	Primary GBM	NCT01310868	Gliadel® and 5-ALA increased cerebrospinal fluid leaks, intracranial hypertension, no survival data published
	Gliadel® wafer	carmustine	Phase II (recruiting)	Metastatic brain tumor	NCT04222062	Not yet reported
	Gliadel® wafer	Carmustine and systemic O6-benzylguanine	Phase II (completed)	Recurrent GBM	NCT00362921	Significant improvement in OS with increase in adverse events of hydrocephalus, CSF leak and CSF/brain infection 180.
	Cerebraca wafer	(Z)-n-butylidenephthalide ((Z)-BP; and Carboxyphenoxypropane-Sebacic Acid Copolymer with adjuvant TMZ	Cerebraca wafer (Phase I & IIa) (recruiting)	Recurrent HGG	NCT03234595	Not yet reported

Abbreviations: PLGA: PEGylated-poly (L-lactic co-glycolic acid); GBM: glioblastoma; TMZ: temozolomide; HGG: high-grade glioma; CSF: cerebrospinal fluid; 5-ALA**:** 5-aminolevulinic acid.
